# Correction of hyperopia with astigmatism following radial keratotomy with daily disposable plus spherical contact lens: a case report

**DOI:** 10.1007/s10792-017-0702-4

**Published:** 2017-08-30

**Authors:** Yun-Wen Chen, Jiahn-Shing Lee, Chiun-Ho Hou, Ken-Kuo Lin

**Affiliations:** 1 Department of Ophthalmology, Kaohsiung Chang Gung Memorial Hospital, Chang Gung University College of Medicine, Taoyuan, Taiwan; 2 Department of Ophthalmology, LinKo Chang Gung Memorial Hospital, Chang Gung University College of Medicine, No.5, Fuxing St., Guishan Dist., Taoyuan City, 333 Taiwan

**Keywords:** Radial keratotomy, Hyperopia, Astigmatism

## Abstract

**Purpose:**

To report the refractive correction in a case of hyperopia and astigmatism following radial keratotomy.

**Methods:**

A case report.

**Results:**

A 47-year-old woman, who had undergone refractive surgery for radial keratotomy in both eyes 22 years before the present study, presented to our clinic with blurred vision. Her best corrected visual acuity, with spectacle correction of +3.50 DS/−1.50 DCX130° in the right eye and +3.75 DS/−1.50 DCX80° in the left eye, was 0.2 logMAR and 0.3 logMAR, respectively. Her keratometric readings were 35.75 D/36.75 D at 74° and 35.25 D/36.25 D at 61°, respectively. Prompted by intolerance to glasses, the patient requested for contact lenses. First, we applied a rigid, gas-permeable contact lens. However, we noted poor fitting due to central corneal flattening. Subsequently, we applied a conventional plus spherical soft contact lens (PSSCL), which is thick in the center and can therefore correct hyperopia and low-grade astigmatism simultaneously. The conventional PSSCL showed slightly inferior decentration, with good movement, and the patient was satisfied with it. After ascertaining the patient’s living habits, we decided that a daily disposable soft contact lens would most meet her needs. The final prescription was a daily disposable PSSCL; the patient was satisfied with her corrected visual acuity of 0.0 logMAR in the right eye and 0.0 logMAR in left eye. Her daily disposable PSSCL-corrected visual acuity was stable during the 10-month follow-up.

**Conclusion:**

For patients displaying hyperopia with astigmatism following radial keratotomy, the PSSCL may confer better corrected visual acuity and acceptability.

## Introduction

Radial keratotomy (RK) is performed using radial incisions in the corneal stroma, producing a wound that is associated with midperipheral bulging of the cornea, compensatory central corneal flattening, and decreased refractive power. For this reason, progressive hyperopia after RK is not uncommon [1, 2]. In fact, in the Prospective Evaluation of Radial Keratotomy (PERK) study, hyperopic shift after RK continued in many patients at an average rate of +0.21 diopters (D) per year for the first 6 months to 2 years after the surgery, and at +0.06 D per year between the second and tenth years after surgery [3, 4]. Therefore, patients who have undergone RK may present with hyperopia many years after the procedure; in such patients, this condition may be related to the corneal incisions, lens aging (including cataract formation), or both [5]. With regard to ocular surgical treatment for RK patients presenting with hyperopia, laser-assisted in situ keratomileusis (LASIK) and surface ablation have been effective [6, 7]. In terms of non-surgical correction methods, contact lenses, including rigid gas-permeable contact lenses (RGPCLs), orthokeratology-designed RGPCLs (OD-RGPCLs), hybrid lenses, and soft contact lenses (SCLs) [8, 9], may improve vision. In the present report, we present a case of hyperopia with astigmatism in a woman who had undergone RK 22 years previously; her vision was well refractive corrected using a plus spherical SCL (PSSCL).

## Case report

A 47-year-old woman visited our outpatient clinic complaining of progressive blurred vision in both eyes. In 1994, she had undergone refractive surgery for RK in both eyes at the age of 25 years, and her preoperative refractive error values were −6.5 D in the right eye and −8.5 D in the left eye. Her uncorrected visual acuity was 0.4 logMAR in both eyes, and her autorefraction (Topcon RM6000, Japan) was +3.75 DS/−1.50 DCX129° in the right eye and +4.00 DS/−1.25 DCX82° in the left eye. Her best corrected visual acuity (BCVA) with spectacle correction of +3.50 DS/−1.50 DCX130° in the right eye and +3.25 DS/−1.00 DCX80° in the left eye was 0.2 logMAR in the right eye and 0.3 logMAR in the left eye. Her keratometric readings were 35.75 D/36.75 D at 74° in the right eye and 35.25 D/36.25 D at 61° in the left eye.

Slit lamp examination revealed a clear cornea with eight radial incisional scars in both eyes; both lenses were clear. Her optic zone after the RK procedure measured 2.4–2.5 mm in the right eye and 2.2–2.3 mm in the left eye. A fundus examination was unremarkable. The intraocular pressure was 19 mmHg in both eyes. The corneal topography of both eyes was obtained using the Oculus Pentacam (Oculus Optikgeräte GmbH, Wetzlar, Germany; Figs. 1, 2); corneal central flattening with refractive powers of 27–30 D was noted (Figs. 3, 4). The patient was therefore diagnosed as having hyperopia with astigmatism following RK.

**Fig. 1:**
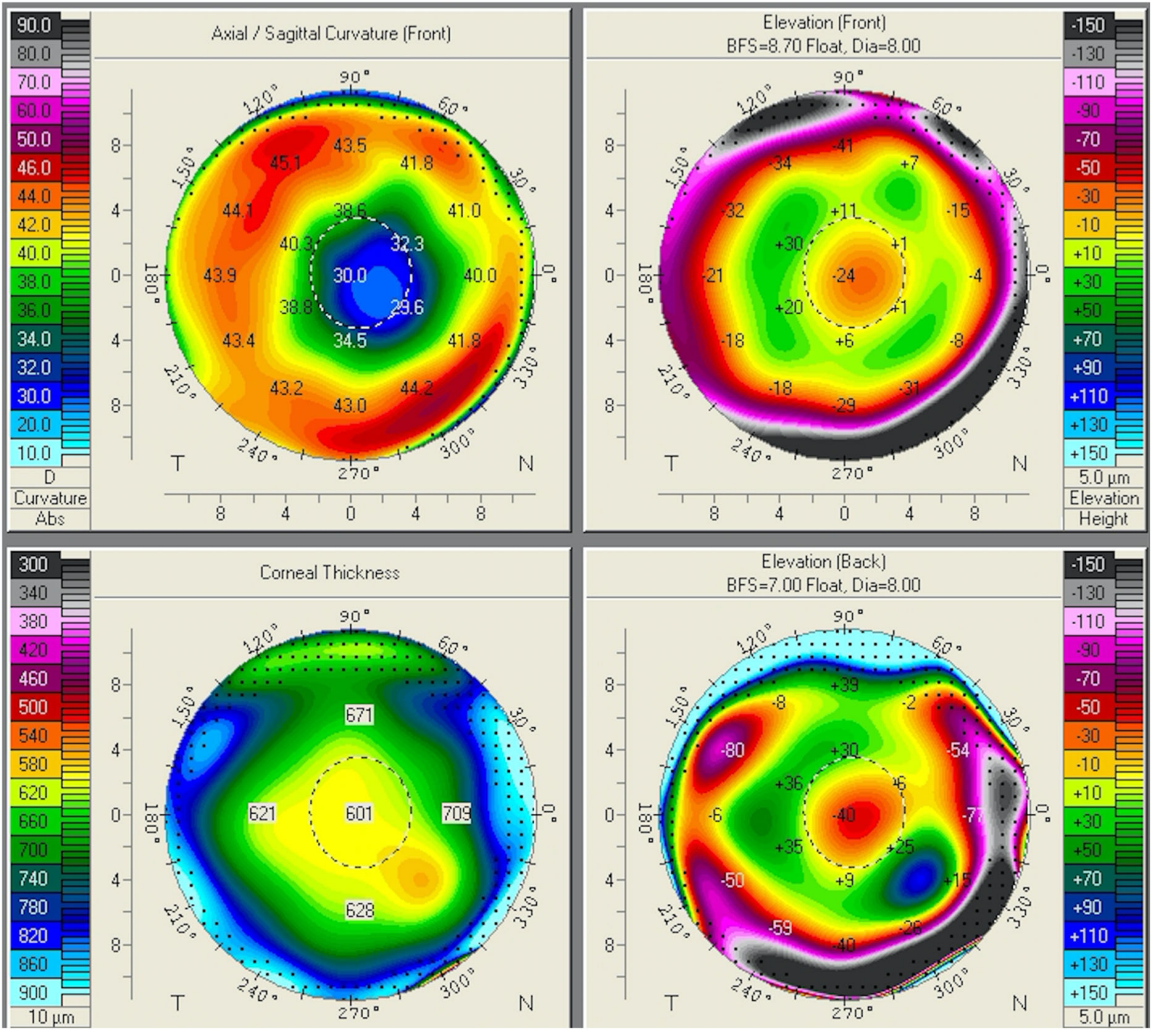
Oculus Pentacam of the patient’s right eye showing corneal curvature map

**Fig. 2:**
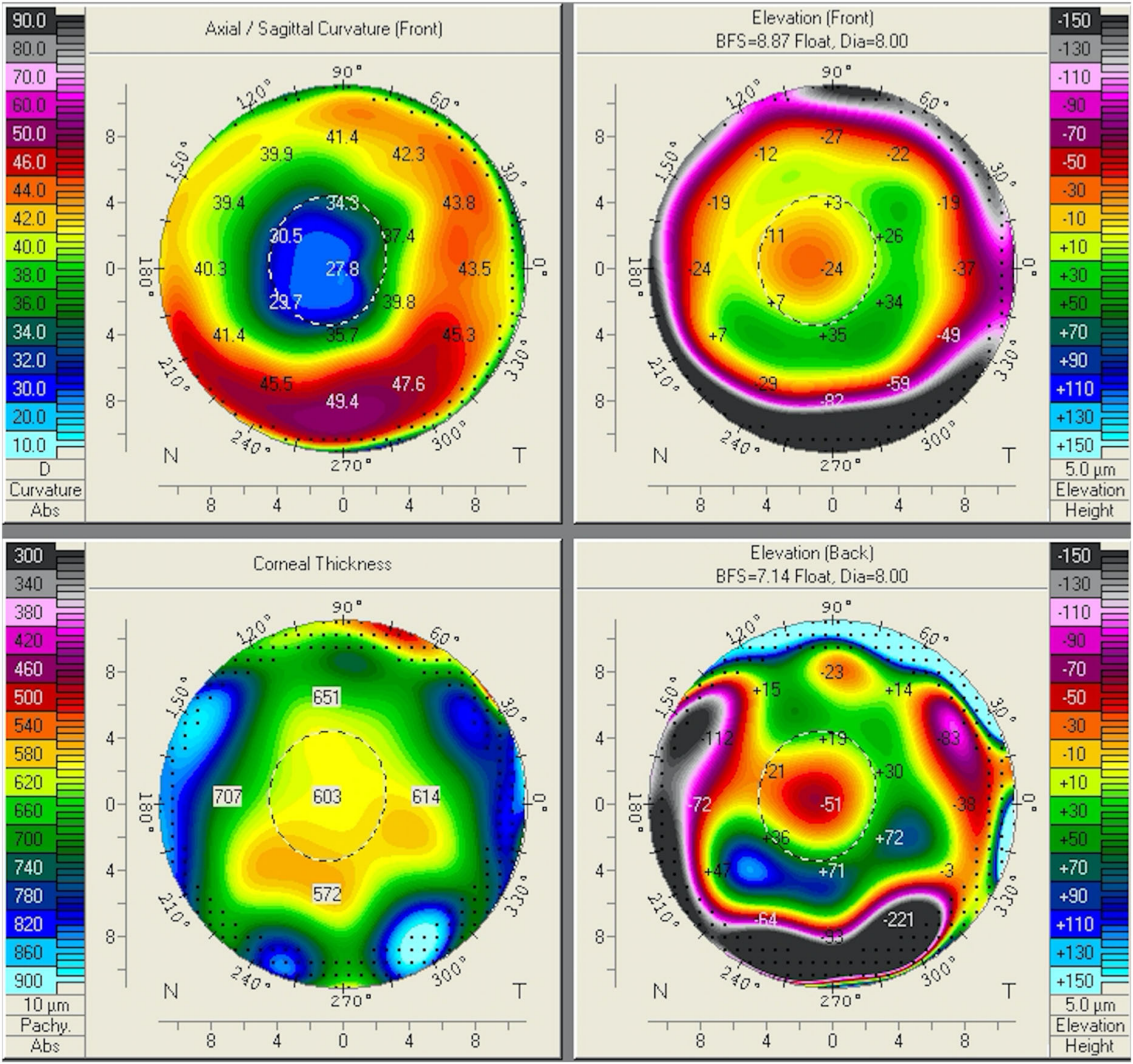
Oculus Pentacam of the patient’s left eye showing corneal curvature map

**Fig. 3:**
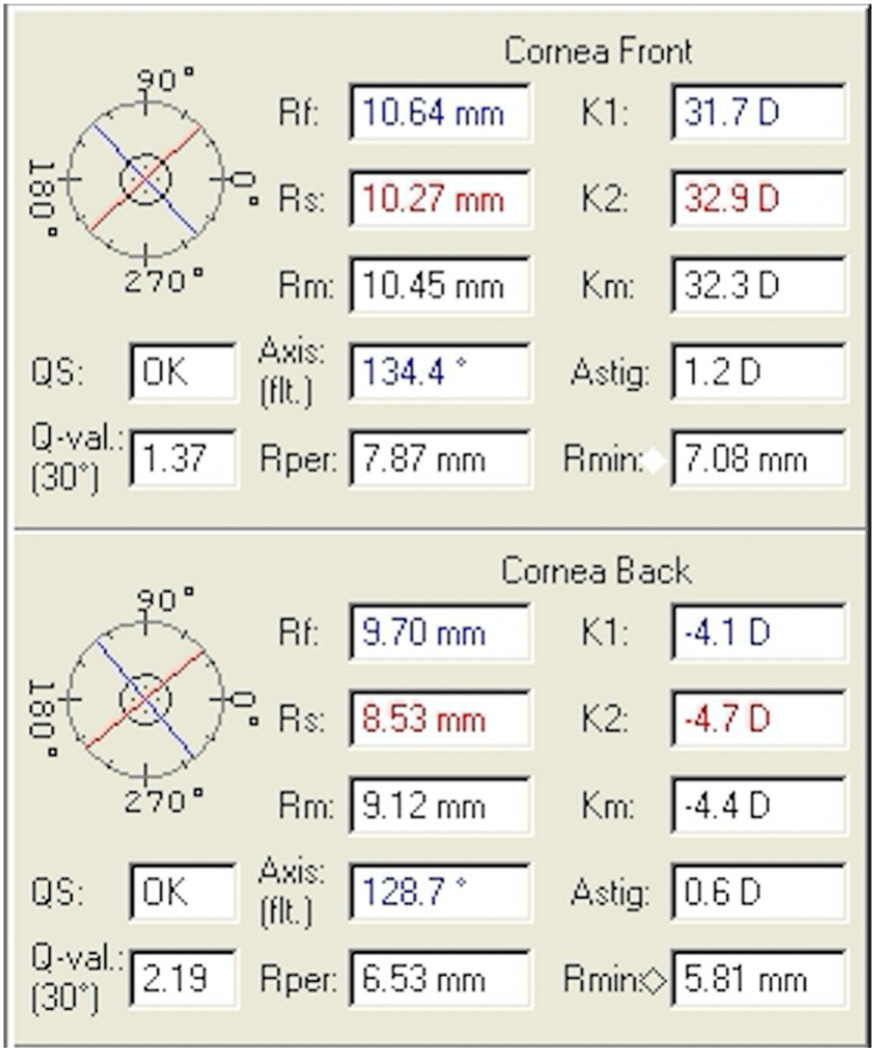
Oculus Pentacam of the patient’s right eye showing corneal parameters

**Fig. 4:**
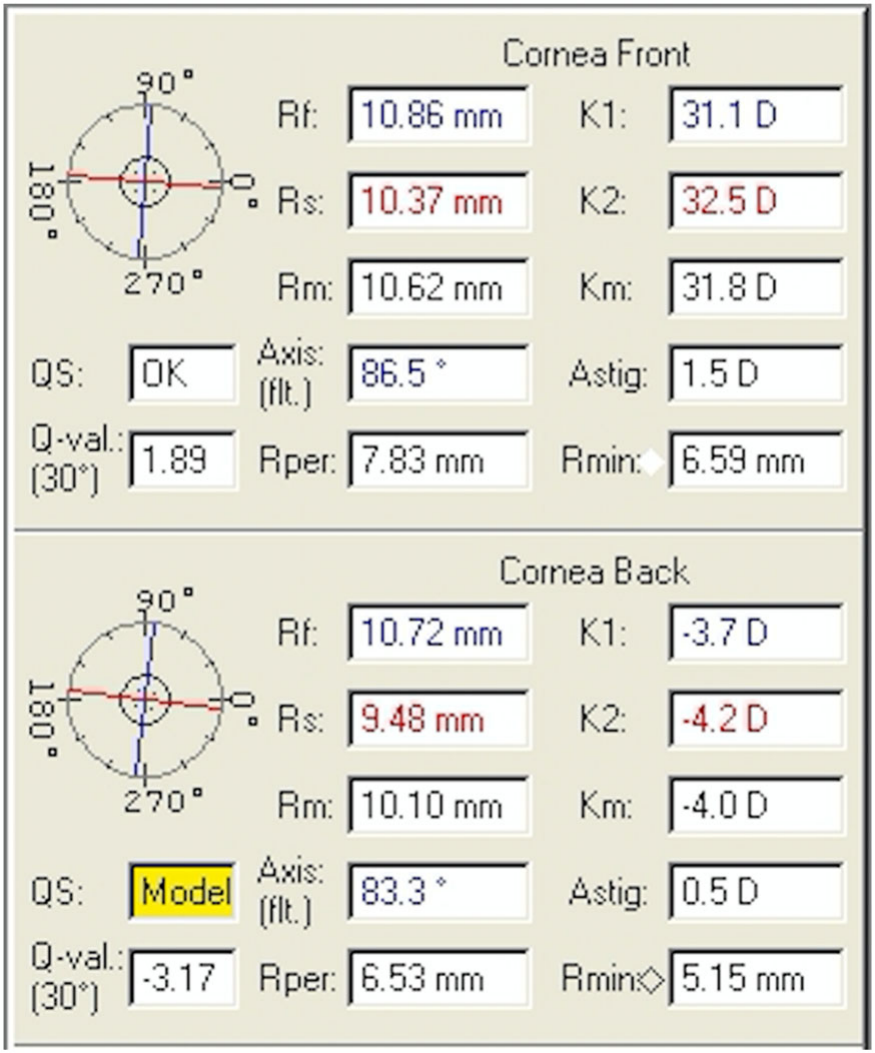
Oculus Pentacam of the patient’s left eye showing corneal parameters

Prompted by an intolerance of glasses, the patient asked for treatment using contact lenses. At first, we applied an RGPCL. However, we noted marked decentration and poor fitting due to central corneal flattening, because the lens did not have a sufficiently flat base curve. Next, we applied a conventional PSSCL, which is thick in the center and can therefore correct hyperopia and low-grade astigmatism simultaneously. The conventional PSSCL showed slightly inferior decentration, with good movement, and the patient was satisfied with her corrected visual acuity. Subsequently, after ascertaining the patient’s living habits, we decided that a daily disposable contact lens would most meet her needs. The final prescription was a Johnson & Johnson^®^ daily disposable PSSCL (+3.50 D/base curve, 8.5 mm/diameter 14.2 mm in the right eye; +3.75 D/base curve 8.5 mm/diameter 14.2 mm in the left eye). The patient was satisfied with her corrected visual acuity of 0.0 logMAR in the right eye and 0.0 logMAR in the left eye. The daily disposable PSSCL-corrected visual acuity was stable during the 10-month follow-up period.

## Discussion

RK is a refractive surgical method for correcting myopia. The procedure involves radial incisions in the paracentral to peripheral cornea; these induce central corneal flattening and reduce myopia. The complications of RK range from the seriously sight-threatening to less severe problems, including corneal perforations, accidental incisions across the visual axis, a decentered clear zone, limbal incisions, visual fluctuations, corneal edema [10], glare [10, 11], and falsely low IOP values [12]. RK may also confer a greater chance of iatrogenic keratoconus, although this speculation remains a matter of debate [13, 14]. Conversely, one case report suggested that corneal stability following RK may protect against keratoconus [13]. The long-term, unintended refractive outcomes of RK noted in the literature include BCVA reduction, irregular astigmatism, over-correction, under-correction, and hyperopic shift [10, 15]. The possible causes of this progressive hyperopic shift are low corneal rigidity and elevated intraocular pressure [16].

Contact lenses, including RGPCLs, SCLs, OD-RGPCLs, and hybrid lenses, may provide a strategy for refractive correction of hyperopia and astigmatism following RK. For instance, Simunovic [8] reported the case of a woman who had previously undergone RK and whose hyperopia was successfully treated using an OD-RGPCL. Traditionally, orthokeratology uses specially designed, reverse-geometry contact lenses that are worn overnight [17–19]. However, in the study described, the patient wore the OD-RGPCL during the daytime. The OD-RGPCL has a central zone that is relatively flat compared with the periphery, and it is therefore suitable to the shape of the post-RK cornea. Similarly, Forister [3] reported the case of a hyperopic patient who had undergone 20-cut and 24-cut RK and who had benefited from an OD-RGPCL for about 1 year after the procedure. However, the lens seemed to be ineffective in the long term. Hybrid lenses have also been reported to confer good results in cases of post-RK hyperopia [8, 9]. For example, Alio et al. [20] reported using hybrid lenses in patients with astigmatism following refractive surgery. However, both OD-RGPCL and hybrid lenses have drawbacks in that they are more expensive and have limited product specifications.

In the present study, after trying RGPCL, we applied a conventional PSSCL, which is thick in the center and which can therefore correct hyperopia and low-grade astigmatism simultaneously. The conventional PSSCL showed slightly inferior decentration, with good movement, and the patient was satisfied with her corrected visual acuity.

The present case suggests that PSSCLs are ideal for refractive correction of hyperopia with astigmatism following RK, because they have a thick center that may better correct both astigmatism and hyperopia. Furthermore, PSSCLs with a thick center can correct the flat central cornea caused by RK; for this reason, they may possess better centration and stability, as well as confer a more stable tear lens.

PSSCLs are easy to apply and are relatively affordable. After ascertaining the living habits of our patient, we decided that a daily disposable SCL might most meet her needs. Ultimately, we prescribed a daily disposable PSSCL, which has all the advantages of the conventional PSSCL and avoids the need for disposable plus toric SCLs, which are unavailable in Taiwan. Our patient showed acceptable fitting and reached a satisfactory corrected visual acuity.

This case study followed the Declaration of Helsinki on medical protocol.

## Conclusion

Patients diagnosed as having hyperopia with astigmatism following RK may benefit from the PSSCL, as the lens confers better corrected visual acuity and acceptability.
